# Partially Automated Method for Localizing Standardized Acupuncture Points on the Heads of Digital Human Models

**DOI:** 10.1155/2015/483805

**Published:** 2015-05-26

**Authors:** Jungdae Kim, Dae-In Kang

**Affiliations:** ^1^Nano Primo Research Center, Advanced Institutes of Convergence Technology, Seoul National University, Suwon 443-270, Republic of Korea; ^2^Pharmacopuncture Medical Research Center, Korean Pharmacopuncture Institute, Seoul 157-801, Republic of Korea

## Abstract

Having modernized imaging tools for precise positioning of acupuncture points over the human body where the traditional therapeutic method is applied is essential. For that reason, we suggest a more systematic positioning method that uses X-ray computer tomographic images to precisely position acupoints. Digital Korean human data were obtained to construct three-dimensional head-skin and skull surface models of six individuals. Depending on the method used to pinpoint the positions of the acupoints, every acupoint was classified into one of three types: anatomical points, proportional points, and morphological points. A computational algorithm and procedure were developed for partial automation of the positioning. The anatomical points were selected by using the structural characteristics of the skin surface and skull. The proportional points were calculated from the positions of the anatomical points. The morphological points were also calculated by using some control points related to the connections between the source and the target models. All the acupoints on the heads of the six individual were displayed on three-dimensional computer graphical image models. This method may be helpful for developing more accurate experimental designs and for providing more quantitative volumetric methods for performing analyses in acupuncture-related research.

## 1. Introduction

Regardless of regional differences between western and eastern medicine, a large amount of medical data has been accumulated over the centuries in the qualitative forms of texts and pictures [1]. Thanks to the fast central process units and vast sizes of computer memories, a new representation of medical knowledge, especially knowledge concerning human anatomy and function, can now be made [2]. Nowadays, the quest to modernize medicine seems to be more demanding on the eastern side than on the western side [3]. As one of the core therapeutic modalities in many Asian countries, acupuncture is still a subject that is actively being studied by with various modernized tools and advanced techniques such as functional magnetic resonance imaging and positron emission tomography [4–6].

In countries such as China, Japan, and Korea, acupuncture has been practiced for more than 2500 years and has now become a global therapeutic method used across the world. Clinical studies have shown promising results for the efficacy of acupuncture in, for example, reducing both postoperative and chemotherapy nausea and vomiting in adults, as well as postoperative dental pain [7]. Although basic research on acupuncture has led to considerable progress over the past decades, its underlying mechanism is still an abstruse subject [8]. Because of the increasing demand for standardization of acupuncture point locations, the World Health Organization Western Pacific Regional Office initiated projects in the early 1990s to reach a consensus on those locations. The WHO presented the general guidelines for acupuncture point locations in the forms of texts and figures, and it stipulated the methodology for locating acupuncture points on the surface of the human body, as well as the locations of 361 standard acupuncture points. This standard established by the WHO may be applied in teaching, research, clinical service, preparation of publications, and academic exchanges involving acupuncture [9].

In more recent studies on acupoint locations, researchers began to use more advanced equipment such as X-ray machines. As a convenient method for locating acupoints, the* cun* measurement methods have been widely used in the practice of acupuncture. However, the traditional* cun* measurement methods have been criticized for their lack of reliability. In one study a comparison of two different location methods was done by using dual-energy X-ray absorptiometry to measure the soft tissue and the bone mass independently [10]. Another study used computed tomography (CT) to provide a metric description of acupuncture points in the lumbar region and to give their relation to individual anatomical landmarks and structures [11]. Another study used X-ray radiography to provide experimental evidence to standardize the location of the acupoint in the hand [12]. Synchrotron radiation phase-contrast X-ray CT was also employed to investigate the three-dimensional (3D) topographic structures of acupuncture points [13]. The results of another study suggested that biomedical information about acupuncture treatment could be visualized in the form of a data-driven 3D acupuncture point system [14].

The accurate localization of acupoints is a key issue in acupuncture research. For more precise scientific research and development on acupuncture therapy, having definitions on how to localize acupoints by using a computer-based pictorial representation of the human body is critical. A method for localizing the acupoints on the head of a virtual body by using segmentation and a 3D visualization of the VOXEL-MAN software system was reported [15]. Our initial research on positioning all the 361 standardized acupuncture points was done with the digital data from a healthy Korean male with a normal body shape [16]. The cross-sectional images generated by X-ray CT were used for generating the 3D virtual models for the bones and the skin's surface of the entire human body. The volumetric 3D acupoint model was developed based on projective 2D descriptions of the standard acupuncture points.

According to the general guidelines suggested by the WHO in the western pacific region, three methods are used for determining acupuncture point locations: the anatomical landmark method, the proportional bone (skeletal) measurement method, and the finger-*cun* measurement method [7]. The anatomical landmark method utilizes some characteristics on the surface of the body that may be fixed or movable, such as protuberances or depressions formed by joints and muscles. The proportional bone (skeletal) measurement method also uses landmarks on the body's surface, that is, primarily joints, to measure the lengths and the widths of various parts of the body. The finger-*cun* measurement method refers to the proportional measurement method for locating acupuncture points based on the size of the fingers of the person to be measured. In some cases, these methods are used together for complementary decisions on the same acupoint.

In this study we developed systematic procedures and algorithms for positioning acupoints on the heads of six individuals. All the acupoints on the head were categorized into one of three types: anatomical points, proportional points, and morphological points. The anatomical acupoints are determined by using corresponding anatomical characteristics from the skin's surface and bone structure. The proportional acupoints are calculated by using prescribed proportional numbers between the anatomical points. Some remaining acupoints only have descriptive definitions for their positions in which case the morphological technique is introduced among the individuals. The traditional methods for the acupoint localization are based on measurements along the surface of the body with* cun*. Here, we do not use* cun* units, which may vary in length for different parts of the body. One of basic assumptions for this study with the head is that the shape of the human head is approximately spherical.

## 2. Materials and Methods

### 2.1. Digital Korean Human Data

Digital Korean CT data for six human beings were obtained from the Korean Institute of Science and Technology Information (KISTI; http://dk.kisti.re.kr/). The CT images of the entire body were taken from three men and three women whose body sizes were measured. Based on the body mass index (BMI), the six individuals were categorized as underweight (18 < BMI < 20 kg/m^2^), normal (20 < BMI < 24 kg/m^2^), overweight (24 < BMI < 26 kg/m^2^), and obese (BMI > 26 kg/m^2^). The genders, ages, heights, and weights of the six individuals are shown in Table 1. A stack of CT images with 512 × 512 pixel resolution was taken from head to toe in 1 mm depth intervals, and the results were saved in the DICOM format.

### 2.2. Procedure for Surface Reconstruction of 3D Human Models

**Figure 1:**
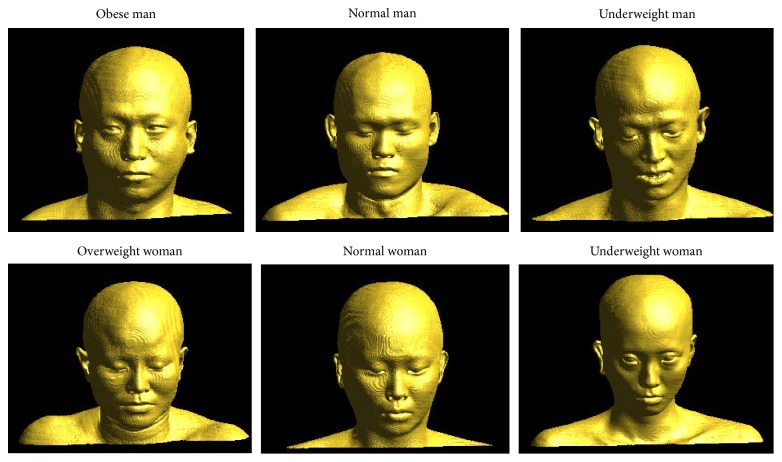
Three-dimensional reconstructed surface models of Korean adults with various body types.

The procedure for reconstructing the surfaces of the skin and the skull from the stack of 2D images of the six individual is basically the same as that used in [16] for a single person with a normal body shape. In brief, first, the DICOM images are converted to 8-bit BMP format because of memory issues. Then, the images for the surfaces of the skin and bone were put into binary format by using the proper threshold values for the pixel intensity, 10 for the skin and 110 for the skull. Unnecessary holes were filled by using a 3D binary dilation subroutine, and boundaries were extracted from the objects in the binary images. The boundary for the skin's surface or for the bone's surface was isolated, and a 3D dataset was made for the skin's surface or for the bone's surface by using the Marching Cube algorithm through the boundary. Actually, the Marching Cube algorithm can be applied to the 8-bit images directly. However, a procedure for binarizing and deleting unnecessary parts is essential in order to obtain model data that are more compact. The 3D image of the surface can be made smoother by averaging the normal vectors at every vertex during the triangulation and by using OpenGL software (https://www.opengl.org/) to present the 3D surface models, as shown in Figure 1, for the skin's surface. The two surface models, one from the skin and the other from the skull of an individual, can be combined in such a way that their centers of mass coincide. The skull can be seen through the skin by computer-graphically giving it some opacity.

### 2.3. Definition for the Reference Frames and the Anatomical Planes

**Figure 2:**
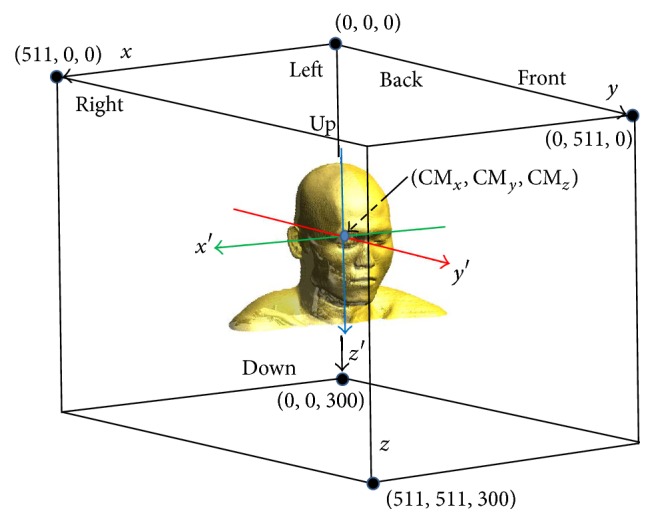
Coordinate systems based on the CT images of the head. For convenient numerical calculations, the *x*-, *y*-, and *z*-axes were chosen to access every voxel from the head skin and skeleton. The model frame (*x*′, *y*′, *z*′) was introduced by translating (*x*, *y*, *z*) to the center of mass of the model and scaling down.

If any point in the 3D digital models is to be described, proper reference frames should be introduced. A natural frame, called the stack frame (*x*, *y*, *z*), can be fixed by using the three indices (*i*, *j*, *k*) along the width, length, and height of the stack of 2D images, as shown in Figure 2. For more a model-dependent frame, the model frame (*x*′, *y*′, *z*′) can be obtained by translating and scaling the stack frame as follows:

(1) $$\begin{array}{c} \left(\;x^{\prime },y^{\prime },z^{\prime }\;\right)=\alpha \left(\;x-{CM}_{x},y-{CM}_{y},z-{CM}_{z}\;\right), \end{array}$$

where the position of the center of mass position for a given stack of binary images, *I*_*ijk*_^bin^, is CM_*x*_ = ∑_*i*,*j*,*k*_ *I*_*ijk*_^bin^ *i*, CM_*y*_ = ∑_*i*,*j*,*k*_ *I*_*ijk*_^bin^ *j*, CM_*z*_ = ∑_*i*,*j*,*k*_ *I*_*ijk*_^bin^ *k*, and we have set *α* = 4/512, for convenience.

**Figure 3:**
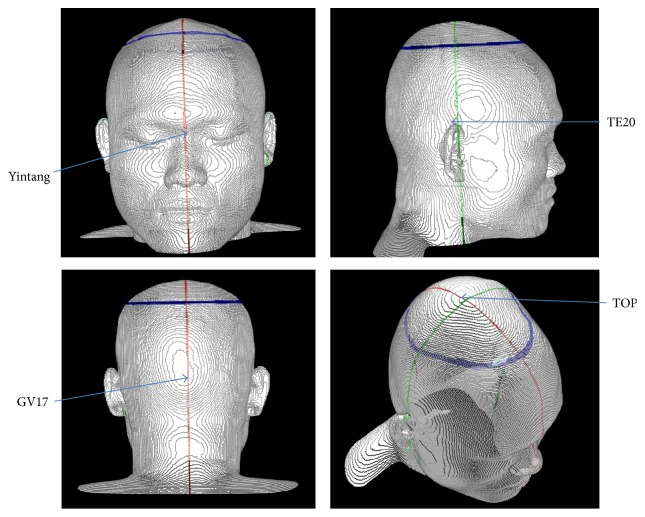
The baselines or planes for the head are determined by using four landmarks: Yintang and GV17 (the red line, the median plane) and TE20 and TOP (the green line, the frontal plane). A transverse plane (the blue line) is determined by using a plane orthogonal to the imaginary line connecting the center of the left and the right TE20 points to the TOP. The four landmarks can be positioned by using their anatomical descriptions on the skin and the skeleton. Yintang is the point between the eyebrow, and TOP is the highest point of the head. The acupoint GV17 is in the depression superior to the external occipital protuberance, and the acupoint TE20 is just superior to the auricular apex on both the left and the right sides.

The anatomical planes for the head can be defined by choosing some obvious points anatomically. The sagittal plane, that is, the midsagittal plane or median plane more precisely, is determined by fixing three points in the head: Yintang (midpoint between the eyebrows), TOP (top of the head), and the acupoint GV17 (external occipital protuberance in the back of the head), as shown in Figure 3. Any point, *P*, which resides in the sagittal plane should satisfy the following equation:

(2) GV17−Yintang×TOP−Yintang·p−Yintang=0,

where all the points in the equation are considered as vectors with triplet values corresponding to the vector's three components and the cross and the dot in the equation mean the cross product and the scalar product, respectively. A coronal plane in the head can be defined by choosing three points on the head: two acupoints TE20 at the auricular apex just above the left and the right ears and the TOP. Any point, *P*, in the coronal plane should satisfy the condition

(3) TE20left−TOP×TE20right−TOP·p−TOP=0.

The transverse plane at any level can be defined in such a way that the plane is perpendicular to the common axis that resides in the sagittal and the coronal planes simultaneously.

### 2.4. Categorization for Acupuncture Points

All the acupoints on the head can be categorized into one of three types depending on how they are located, as shown in Table 2. In addition to the standard acupuncture points, three more points, Pupil, Yintang, and TOP, are introduced for positioning the acupoints. The anatomical acupoints have anatomical descriptions that are more or less clear based on the structures of the skin or the skull. Brief descriptions on the acupoints on the head are presented in Table 3. The corresponding surface models for their positioning are also given in the table.

For the proportional acupoints, we first introduce the midpoint between the left and the right TE20 as follows:

(4) $$\begin{array}{c} TE20CP\equiv \frac{\left({TE20}_{left}+{TE20}_{right}\right)}{2}. \end{array}$$

This imaginary point is introduced just for arithmetic convenience. In order to obtain the conditions for positioning the proportional acupoints, let us define the angle *θ*_0_ made by the three points Yintang, TE20CP, and GV17, with the vertex point being TE20CP, as follows:

(5) $$\begin{array}{c} \theta_{0}=\cos^{-1}\frac{\left(Yintang-TE20CP\right)\cdot \left(GV17-TE20CP\right)}{\left|Yintang-TE20CP\right|\left|GV17-TE20CP\right|}. \end{array}$$

We can also calculate two angles depending on the point *P*, which is one of the voxels for the surface of the skin on the head. If the point *P* is on the skin's surface in the sagittal plane, we define the angle as follows:

(6) $$\begin{array}{c} \theta_{1}=\cos^{-1}\frac{\left(p-TE20CP\right)\cdot \left(GV17-TE20CP\right)}{\left|p-TE20CP\right|\left|GV17-TE20CP\right|}. \end{array}$$

If the point *P* is on the skin's surface in the transverse plane, we define the angle as follows:

(7) $$\begin{array}{c} \theta_{2}=\cos^{-1}\frac{\left(p-TE20CP\right)\cdot \left(GV24-TE20CP\right)}{\left|p-TE20CP\right|\left|GV24-TE20CP\right|}. \end{array}$$

**Figure 4:**
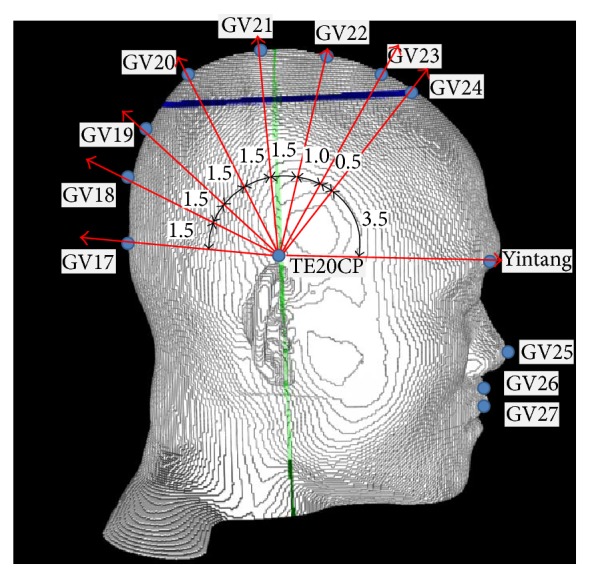
Some proportional acupoints from GV18 to GV24 on the midsagittal plane that were obtained by dividing the angle between GV17 and Yintang by appropriate proportionality constants based on the guidelines of the WHO for standard acupuncture positioning.

The conditions for the proportional acupoints are displayed in Table 4, and the pictorial descriptions of the conditions are shown in Figure 4.

**Figure 5:**
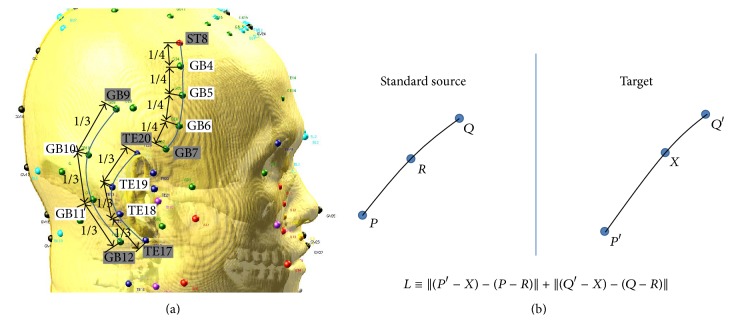
Morphological acupoints on the right side of the head (a) and the distance formula for the unknown position *X* with the known positions *P*′, *Q*′ from the target model and the *P*, *Q*, and *R* from the standard source model. The position *X* is adjusted in such a way that the distance *L* is minimized.

According the standard acupuncture positioning in [9], some acupoints are described on the curved lines between two acupoints, as shown in Figure 5(a) and Table 5. For example, GB4, GB5, and GB6 are positioned along the curved line between ST8 and GB7. In this case, we use the morphological method for positioning the acupoints GB4, GB5, and GB6 by using the previously-known points ST8 and GB7 as two control points. Precise information about positioning the morphological acupoints should be prepared from a “standard” source. We took that “standard” source to be the 3D model of the normal man, as displayed in Figure 5(a), and we obtained the positions of the anatomical and proportional acupoints in advance.  Figure 5(b) shows that the target point *X* between the two control points *P*′ and *Q*′ can be calculated in such a way that the sum of the distances between the two control points is minimized compared to the distances of the two control points from the standard source.

## 3. Results

**Figure 6:**
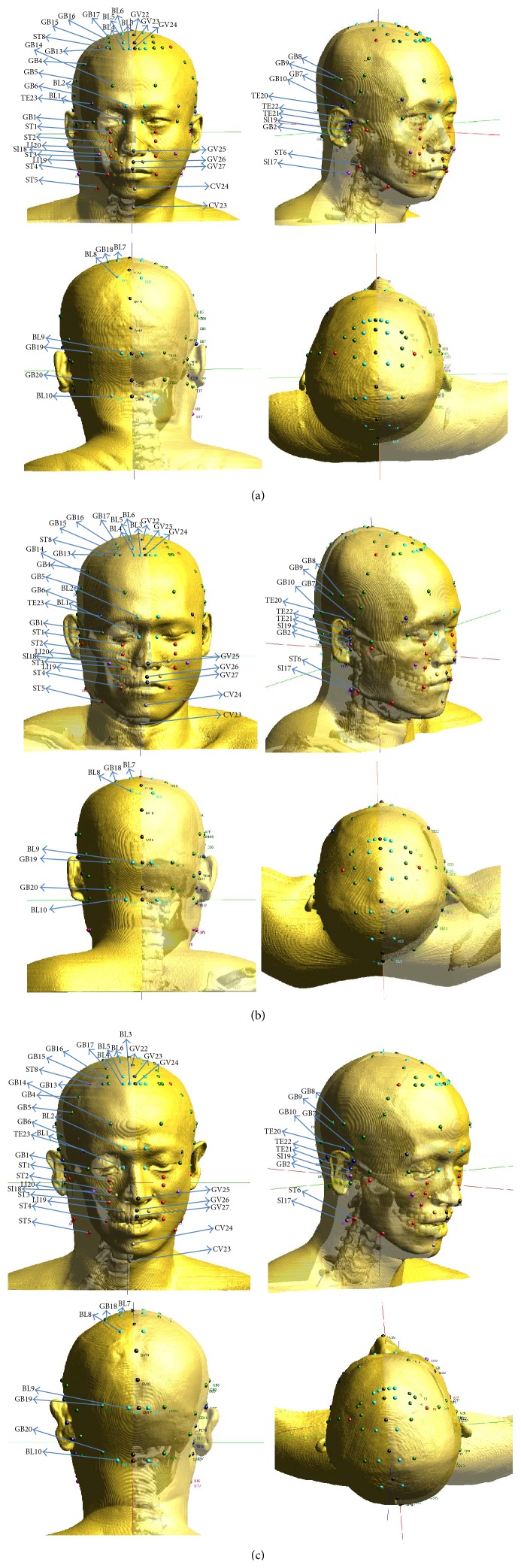
Various views of the 3D model of an (a) obese, a (b) normal, and an (c) underweight man with positioned acupoints on their heads.

All the acupoints on the head above the neck were calculated following the above procedure. We positioned the acupoints on the head models taken from the 3D digital CT images. Figures 6(a), 6(b), and 6(c) show various views of the heads of the obese, the normal, and the underweight man, respectively. The total of 65 standard acupoints on the head could be classified into 34 anatomical points, 24 proportional points, and 7 morphological points. The positions of the anatomical acupoints were described, and those descriptions are summarized in Table 3. Based on the descriptions, the anatomical acupoints were positioned manually, and their positions were saved for further calculations on positioning the proportional and the morphological acupoints.

The analytic and pictorial descriptions of the proportional acupoints are presented in Table 4 and Figure 4, respectively. If we assume that the shape of the surface of the head is spherical, the distance between consecutive acupoints in the midsagittal planes can be approximately determined by using the dividing the angle between the two points with a vertex into smaller angles. The acupoints from GV17 to GV24, for example, are positioned by dividing the angle between the GV17 and Yintang with the vertex TE20CP into smaller subangles with proportional values, as shown in Figure 4. In the numerical code for positioning the proportional acupoints, proper small intervals are introduced for every condition in Table 4. If a pixel point on the skin's surface satisfied the condition within the interval, that pixel point was included, and an average point, which was taken to be the position for the proportional acupoint, was obtained. Traditionally, all the acupoints are classified according to the meridians to which they belong.

**Figure 7:**
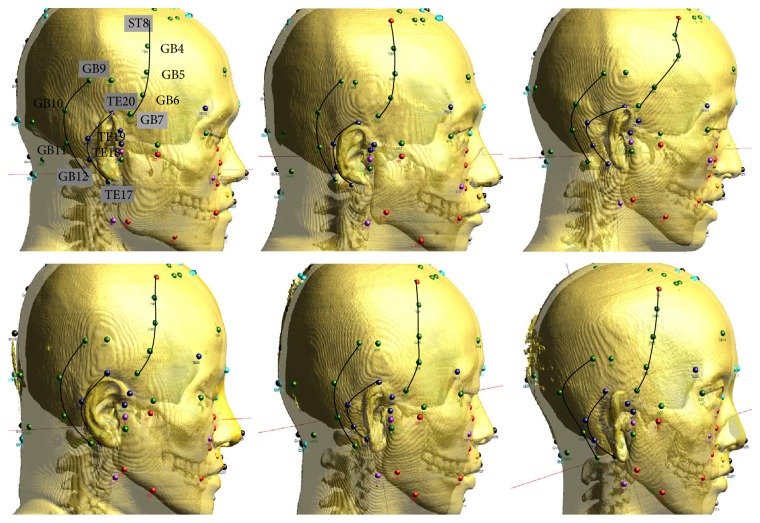
Morphological acupoints and the lines connecting the two control points through them. The upper left corner shows the morphological acupoints on the head of anormal man; they exactly match the same positionings on our “standard” source model for the morphological technique. In the other models, the shapes of the curves are shown to be slightly different, depending on the individuals.

The results for the morphological acupoints for the six subjects are shown in Figure 7. The figures are displayed for the right sides of the heads, and the three lines in each head connect the two control points with the morphological acupoints along the curves. The morphological acupoints GB4, GB5, and GB6, for example, reside on the curve connecting the control points of ST8 and GB7. Our computer code for the morphological technique has been checked to start positioning the morphological acupoints with the model of the normal man (the panel in the upper left corner in Figure 7). By adopting the model of the normal man as the standard source model, we confirmed that the source points precisely match the target points in the case of the normal man. The same code was applied to the other models to obtain the morphological acupoints. Figure 7 shows lines with slightly different shapes that depend on the surface structures of the heads of the other two men (the obese man and the underweight man) and those of the three women (the overweight woman, the normal woman, and the underweight woman).

## 4. Discussion 

Recently, considerable efforts have been made to understand the characteristics of acupuncture points [17, 18]. In our previous study, acupuncture point positioning for the entire body of a single person was done by using the 3D digital model of the normal man. The reference points or the landmarks were positioned based on the standard descriptions of the acupoints, and the formulae for the proportionalities between the acupoints and the reference points were presented everywhere on the body. We found that the 37% of the 361 standardized acupoints on the entire body were automatically linked to the reference points. The reference points accounted for 11% of the 361 acupoints, and the remaining acupoints (52%) were positioned point-by-point by using 3D computer graphics libraries [16]. In this study, we increased the number of subjects to six and confined the positioning area to their heads. For the locations of some acupoints, we used the morphological technique in which topographical constraints between acupoints were considered among the individuals. Many morphological approaches to handling medical image data have developed for use in basic research, as well as in clinical studies, in various fields [19–22].

Currently, substantial interrater variability in acupoint location and intrarater variability within the clinical setting exist because the optimal location of the point therapeutically may deviate from the location of the standard textbook point and may vary from treatment to treatment. That is, in addition to the anatomical, proportional, and morphological considerations for locating a point, a clinical palpatory consideration may also exist, which may involve modifying the textbook location of the point based upon the texture or the tension of the tissue as detected by the practitioner or based upon the “Ashi” tenderness felt by the patient. Our approach can also accommodate the localization of any additional points on the body that might be clinically relevant for treatment.

The traditional methods for acupoint localization are based on measurements along the surface of a body with* cun*, the traditional Chinese measure, which varies in length for different parts of the body and for different directions of the longitude (vertical) and latitude (horizontal). With a contrasting approach to this work, authors of [15] introduced three projection planes on the head for a 2D acupoint description system. For a definite viewing direction, all visible points on the body's surface have one projection plane that is perpendicular to that viewing direction, and the visible points have corresponding projection points in that projection plane. Each projection point may be back-projected to the body's surface to obtain the corresponding 3D coordinates. This project matching between the 2D and the 3D images is necessary to satisfy the traditional descriptions on positioning acupoints.

In conclusion, we propose a partially automated method and procedure for localizing the standardized acupuncture points on the heads of 3D digital CT human models taken from six living individuals. In the future, we expect to be able to develop for practical clinical use a fully automated method for positioning acupuncture points on the entire body of an individual.

## Figures and Tables

**Table 1:** Characteristics of six subjects for the 3D digital CT images.

Subject	Gender	Age (years)	Height (cm)	Weight (kg)	BMI (kg/m^2^)	Category
1	Male	26	170	66	22.84	Normal
2	Male	37	175	59	19.27	Underweight
3	Male	39	170	75	27.34	Obese
4	Female	30	158	55	20.20	Normal
5	Female	28	161	48	18.52	Underweight
6	Female	43	162	64	24.01	Overweight

**Table 2:** Categorized acupoints on the head. The acronyms for the acupoint names come from the names of meridians: GV (governor vessel), GB (gall bladder), ST (stomach), TE (triple energizer), BL (bladder), CV (conception vessel), LI (large intestine), and SI (small intestine). The values in the parentheses are the total numbers of points in the categories. ^*∗*^These points are not standard acupuncture points but are introduced for convenience.

Category	Acupoints on the head
Anatomical points (34)	GV15, GV16, GV17, GV25, GV26, GV27 (6)
	GB1, GB2, GB3, GB7, GB8, GB9, GB12, GB20 (8)
	ST1, ST2, ST3, ST4, ST5, ST6, ST7 (7)
	TE17, TE20, TE21, TE22, TE23 (5)
	BL1, BL2 (2)
	CV23, CV24 (2)
	LI19, LI20 (2)
	SI17, SI18 (2)
	Pupil^*∗*^, Yintang^*∗*^, TOP^*∗*^ (3)

Proportional points (24)	GV18, GV19, GV20, GV21, GV22, GV23, GV24 (7)
	GB13, GB14, GB15, GB16, GB17, GB18, GB19 (7)
	ST8 (1)
	BL3, BL4, BL5, BL6, BL7, BL8, BL9, BL10 (8)
	SI19 (1)

Morphological points (7)	GB4, GB5, GB6, GB10, GB11 (5)
	TE18, TE19 (2)

**Table 3:** Descriptions of the locations of the anatomical acupuncture points and landmarks. ^*∗*^These landmarks are additionally positioned for localization of the acupoints.

Acupoints/landmarks	Surface models	Description of the position for the landmark
GV15	Bone	In the depression superior to the spinous process of the second cervical vertebra (C2) on the posterior median line
GV16	Bone	Directly inferior to the external occipital protuberance
GV17	Bone	External occipital protuberance
GV25	Skin	Tip of the nose
GV26	Skin	Midpoint of the philtrum midline
GV27	Skin	Midpoint of the tubercle of the upper lip

GB1	Skin and bone	Outer canthus of the eye
GB2	Bone	Depression between the intertragic notch and the condylar process of the mandible
GB3	Bone	Depression superior to the midpoint of the zygomatic arch
GB7	Skin	Junction of the vertical line of the posterior border of the temple hairline and the horizontal line of the apex of the auricle
GB8	Skin	Directly superior to the auricular apex
GB9	Skin	Directly superior to the posterior border of the auricular root
GB12	Bone	Depression posteroinferior to the mastoid process
GB20	Bone	Inferior to the occipital bone, in the depression between the origins of the sternocleidomastoid and the trapezius muscles

ST1	Skin and bone	Between the eyeball and the infraorbital margin, directly inferior to the pupil
ST2	Bone	In the infraorbital foramen
ST3	Skin	Directly inferior to the pupil, at the same level as the inferior border of the ala of the nose
ST4	Skin	The angle of the mouth
ST5	Bone	Anterior to the angle of the mandible, in the depression anterior to the masseter attachment
ST6	Bone	Angle of the mandible
ST7	Bone	Depression between the midpoint of the inferior border of the zygomatic arch and the mandibular notch

TE17	Skin	Posterior to the ear lobe, in the depression anterior to the inferior end of the mastoid process
TE20	Skin	Auricular apex
TE21	Skin and bone	In the depression between the supratragic notch and the condylar process of the mandible
TE22	Skin	Anterior to the auricular root, posterior to the superficial temporal artery
TE23	Skin	In the depression at the lateral end of the eyebrow (it is superior to GB1)

BL1	Skin and bone	In the depression between the superomedial parts of the inner canthus of the eye and the medial wall of the orbit
BL2	Skin	In the depression at the medial end of the eyebrow

CV23	Bone	In the anterior region of the neck, superior to the superior border of the thyroid cartilage, in the depression superior to the hyoid bone, on the anterior median line
CV24	Skin	In the depression in the center of the mentolabial sulcus

LI19	Skin	At the same level as the midpoint of the philtrum
LI20	Skin	In the nasolabial sulcus, at the same level as the midpoint of the lateral border of the ala of the nose

SI17	Bone	Posterior to the angle of the mandible, in the depression anterior to the sternocleidomastoid muscle
SI18	Skin and bone	Inferior to the zygomatic bone, in the depression directly inferior to the outer canthus of the eye

Pupil^*∗*^	Skin	Center line of the pupil
Yintang^*∗*^	Skin	Midpoint between the eyebrows
TOP^*∗*^	Skin	Top of the head

**Table 4:** Proportional acupoints. The left and the right sides of the acupoints are determined by the positions of *P* whether the acupoints are located on the left and the right side of the *yz* plane, respectively. ^†^The point AUX is introduced for arithmetic convenience.

Conditions	Calculated points
$\frac{\theta_{1}}{\theta_{0}}=\frac{1.5}{12.5}$	*P* = GV18
$\frac{\theta_{1}}{\theta_{0}}=\frac{3.0}{12.5}$	*P* = GV19
$\frac{\theta_{1}}{\theta_{0}}=\frac{4.5}{12.5}$	*P* = GV20
$\frac{\theta_{1}}{\theta_{0}}=\frac{6.0}{12.5}$	*P* = GV21
$\frac{\theta_{1}}{\theta_{0}}=\frac{7.5}{12.5}$	*P* = GV22
$\frac{\theta_{1}}{\theta_{0}}=\frac{8.5}{12.5}$	*P* = GV23
$\frac{\theta_{1}}{\theta_{0}}=\frac{9.0}{12.5}$	*P* = GV24
$\frac{\theta_{1}}{\theta_{0}}=\frac{11.0}{12.5}$	*P* = AUX^†^

$\frac{\theta_{2}}{\theta_{0}}=\frac{2.25}{12.5}$	*P* = ST8
$\frac{\theta_{2}}{\theta_{0}}=\frac{1.5}{12.5}$	*P* = GB13
*P*_*x*_ = Pupil_*x*_, *P*_*z*_ = AUX_*z*_	*P* = GB14
$\frac{\theta_{2}}{\theta_{0}}=\frac{0.25}{12.5}$	*P* = BL3
$\frac{\theta_{2}}{\theta_{0}}=\frac{0.5}{12.5}$	*P* = BL4

*P*_*x*_ = Pupil_*x*_, *P*_*z*_ = GV24_*z*_	*P* = GB15
*P*_*x*_ = Pupil_*x*_, *P*_*z*_ = GV23_*z*_	*P* = GB16
$P_{x}={\text{Pupil}}_{x},P_{z}=\frac{1}{2}*\text{GV}22_{z}+\frac{1}{2}*\text{GV}23_{z}$	*P* = GB17

*P*_*x*_ = BL4_*x*_, *P*_*z*_ = GV23_*z*_	*P* = BL5
$P_{x}=\text{BL}4_{x},P_{y}=\frac{1}{3}*\text{GV}21_{y}+\frac{2}{3}*\text{GV}22_{y}$	*P* = BL6
$P_{x}=\text{BL}4_{x},P_{y}=\frac{1}{3}*\text{GV}20_{y}+\frac{2}{3}*\text{GV}21_{y}$	*P* = BL7
$P_{x}=\text{BL}4_{x},P_{y}=\frac{1}{3}*\text{GV}19_{y}+\frac{2}{3}*\text{GV}20_{y}$	*P* = BL8
$P_{x}=\text{GV}17_{x}-\frac{1.3}{1.5}*\left(\text{BL}4_{x}-\text{GV}24_{x}\right)$, *P*_*z*_ = GV17_*z*_	*P* = BL9
*P*_*x*_ = GB17_*x*_, *P*_*z*_ = BL7_*z*_	*P* = GB18
*P*_*x*_ = GB20_*x*_, *P*_*z*_ = GV17_*z*_	*P* = GB19
$P_{x}=\frac{1}{2}*\left(\text{GV}16_{x}+\text{GB}20_{x}\right)$, *P*_*z*_ = GV15_*z*_	*P* = BL10
$P=\frac{1}{2}*\left(\text{TE}21+\text{GB}2\right)$	*P* = SI19

**Table 5:** Seven acupoints categorized as morphological points with the two corresponding control points. The values in the parentheses are the “standard positions” of the acupoints on the left and the right sides of the head taken from the 3D digital model of the normal man. These values were used as the standard for calculating the morphological acupoints in the other models.

Acupoints		Control points	
GB4	Left: (−0.539, 0.274, −0.912)		
	Right: (0.560, 0.274, −0.912)	ST8	Left: (−0.447, 0.278, −1.026)
GB5	Left: (−0.610, 0.272, −0.759)		Right: (0.457, 0.278, −1.026)
	Right: (0.628, 0.272, −0.759)	GB7	Left: (−0.641, 0.155, −0.474)
GB6	Left: (−0.639, 0.239, −0.594)		Right: (0.651, 0.155, −0.474)
	Right: (0.655, 0.239, −0.594)		

GB10	Left: (−0.584, −0.266, −0.467)	GB9	Left: (−0.641, −0.089, −0.714)
	Right: (0.541, −0.266, −0.467)		Right: (0.613, −0.089, −0.714)
GB11	Left: (−0.556, −0.266, −0.220)	GB12	Left: (−0.529, −0.138, 0.027)
	Right: (0.541, −0.266, −0.220)		Right: (0.529, −0.138, 0.027)

TE18	Left: (−0.555, −0.159, −0.128)	TE17	Left: (−0.546, −0.004, 0.027)
	Right: (0.555, −0.134, −0.128)		Right: (0.566, −0.004, 0.027)
TE19	Left: (−0.604, −0.159, −0.284),	TE20	Left: (−0.628, −0.004, −0.459),
	Right: (0.585, −0.159, −0.284)		Right: (0.623, −0.004, −0.459)
